# Idiopathic Atrophoderma of Pasini and Pierini: Case report and literature review

**DOI:** 10.1002/ccr3.1958

**Published:** 2018-12-18

**Authors:** Anastasiya Muntyanu, Margaret Redpath, Osama Roshdy, Abdulhadi Jfri

**Affiliations:** ^1^ Faculty of Medicine University of Ottawa Ottawa Ontario Canada; ^2^ Division of Pathology Jewish General Hospital Montréal Québec Canada; ^3^ Division of Dermatology Jewish General Hospital Montreal Québec Canada; ^4^ Division of Dermatology McGill University Health Centre Montreal Québec Canada

**Keywords:** Atrophoderma, Atrophoderma of Pasini and Pierini

## Abstract

Idiopathic Atrophoderma of Pasini and Pierini should be considered on the differential in a patient presenting with an asymptomatic atrophic plaque on the skin. Differentiation from Linear Atrophoderma of Moulin and morphea remains a challenge; however, features of the presentation and tissue biopsy can help establish the diagnosis.

## 1 INTRODUCTION

Idiopathic Atrophoderma of Pasini and Pierini (IAPP) is a rare skin disease thought to affect dermal collagen organization resulting in atrophy. We present a 23‐year‐old man with an atrophic hyperpigmented plaque, normal laboratory findings, and perivascular lymphohistiocytic infiltrate on histopathology. Furthermore, we reviewed the current literature pertinent to this case.

Idiopathic Atrophoderma of Pasini and Pierini is a rare skin disease which is thought to affect dermal collagen organization resulting in dermal atrophy. Pasini first described this condition in 1923 as “progressive idiopathic atrophoderma”^1^ and later Pierini suggested its link to morphea.^2^ Finally, in 1958, Canizares et al coined the term Atrophoderma of Pasini and Pierini (APP) and identified unique features in comparison to morphea.^3^ There are <100 cases of this condition reported in the literature to date. The usual presentation is thought to occur most commonly in Caucasian Europeans in the second to third decade with a female predominance of 6:1.^4^

The classic clinical manifestations include hyperpigmented or hypopigmented, depressed areas of skin with classic “cliff‐drop” borders.^5^ The distribution most often includes the trunk and then progresses to include the chest, arms, and abdomen. There may be associated pain, pruritus, or even paresthesia.^4^

The causal or associated factors have been challenging to elucidate. Some reports have linked IAPP to Borrelia burgdorferi infection.^6^ In one study, IgG antibodies were positive in 53% of patients with IAPP as compared to 14% of control subjects.^6^ Some have described familial atrophoderma; however, a genetic link still needs to be confirmed.^4^ Additionally, concordance between siblings further suggests a possible genetic cause.^7^ In two reports, there was an association with a neoplastic process such as extramedullary plasmacytoma^8^ and papillary cancer of the thyroid gland.^9^ Thus, further collection of the literature about IAPP is necessary to be able to better define the predisposing factors.

The objective of this case report is to demonstrate the clinical and histopathological features of IAPP and discuss the differential diagnosis.

## 2 CASE HISTORY

**Figure 1:**
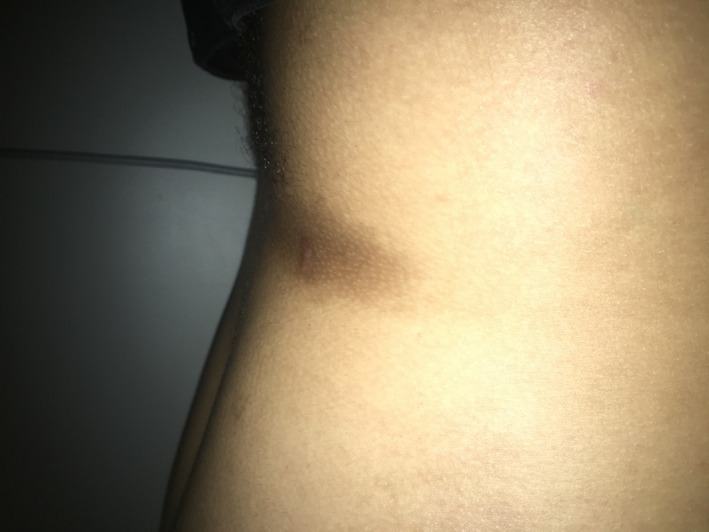
Hyperpigmented atrophic plaque with “cliff‐drop” edge on the lower back

**Figure 2:**
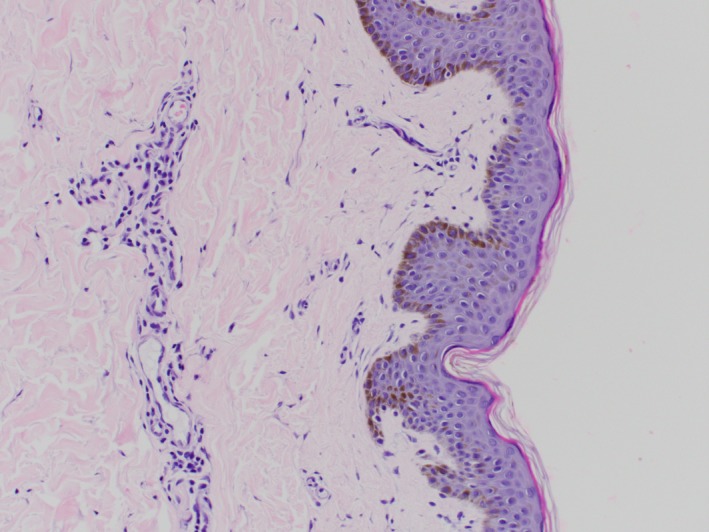
Low power

**Figure 3:**
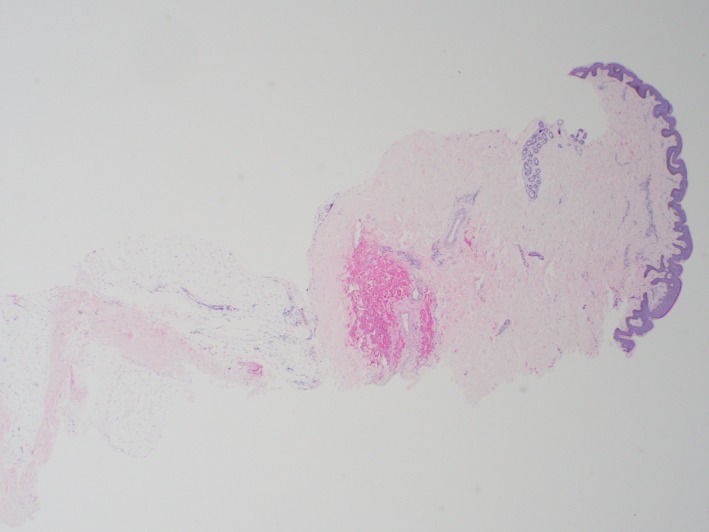
High power: (magnification ×) Hyperpigmentation of the basal layer is present with dermal edema and a mild perivascular lymphohistiocytic infiltrate with plasma cells

**Figure 4:**
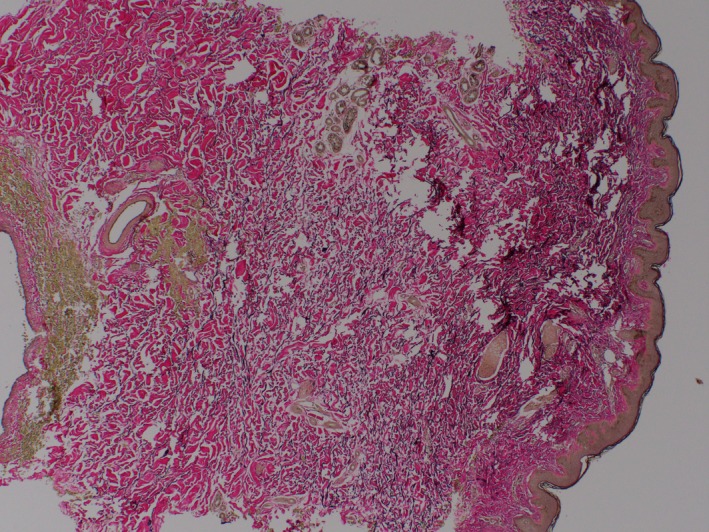
Elastic fiber stain

A 23‐year‐old male, originally from Morocco and otherwise healthy, presented to the Dermatology clinic with a 3‐year history of abnormal atrophic skin texture on the lower back. The lesion was slowly expanding over months initially and then plateaued within 2 months prior to presentation. He denied pain, pruritus, paresthesia, or any other symptoms. He did not use any topical or systemic treatments for this lesion. He was not on any medications and did not have a history of food or drug allergy. He denied any family history of similar lesions. Review of systems was unremarkable. He traveled frequently to his home country, Morocco. On exam, there was a 4 × 11 cm well‐demarcated atrophic hyperpigmented plaque on the lower back (Figure 1), with normal surrounding skin. A 4‐mm punch biopsy was taken from the lesion that showed dermal edema with a mild perivascular lymphohistiocytic infiltrate with plasma cells and normal collagen (Figures 2, 3, 4). Laboratories were requested for Lyme screening serology (IgG antibody), antinuclear antibody, and rheumatoid factor, and the results were negative. The patient was not offered a treatment.

## 3 DISCUSSION

Idiopathic Atrophoderma of Pasini and Pierini can present as single or multiple lesions with the back being involved in most of the reported cases in the literature (Table 1). Several different modalities have been employed to try and better identify the characteristic features of the disease and compare to other similar presentations. On histology, the most common finding is a decrease in thickness of the dermis and absence of sclerosis.^10^ Comparison with normal skin may be required to discern that the dermis is atrophic. Additionally, there is a presence of interstitial edema and a mild perivascular infiltrate consisting of lymphocytes and histiocytes.^4^ The sweat glands, pilosebaceous units, and appendages all have a normal appearance.^11^ The study by Vieira‐Damiani et al utilized multiphoton microscopy and found no difference in optical density of collagen or elastic fibers in affected skin and normal skin.^11^ Interestingly, horizontal collagen fibers in the lesion were increased toward the lower portion of the dermis while elastic fibers depicted greater disorganization within the upper dermis. Thus, there is no difference in the content of collagen and elastic tissue but rather there is a difference in the organization. The epidermis generally remains normal, but slight atrophy with flattened rete ridges could be seen.^4^ An ultrasound analysis was conducted to try and further identify unique features of the disease, and it was found that the dermal thickness on the sonogram was decreased by 46%‐55% and hypodermis by 10%‐18% in affected skin compared to normal skin in the same patients. However, there was no associated change in echogenicity.^12^

**Table 1:** Comparison between the different characteristics of reported cases with Idiopathic atrophoderma of Pasini and Perini (IAPP)

Age/study	Gender	Number of lesions (sites)	Investigation	Histopathology	Treatment
34^8^	M	Multiple (Axillae and bilateral trunk)	Elevated erythrocyte sedimentation rate (ESR) and C‐reactive protein (CRP)	Normal epidermis, a mild mononuclear perivascular infiltrate and homogenization of collagen fibers with unaffected adnexal structures in the dermis. The collagen fibers stained blue with Masson trichrome staining	Prednisolone 40 mg/d for 7 d, with 2w taper. No improvement. Oral doxycycline 200 mg/d for 14 d with potent topical corticosteroid ointment without improvement
37^23^	F	Single (Chest)	None	Slightly atrophic epidermis. Collagen bundles were homogenized and clumped in the mid‐dermis. Orcein staining showed fragmented elastic fibers near a pilosebaceous follicle	None
10^24^	F	Multiple (Upper limb and thigh)	None	Mild inflammatory changes with perivascular and focally interstitial lymphoid infiltrates without any obvious signs of sclerosis	Month long trials of topical steroids, vitamin D analogues, retinoid, and hydroquinone over 5 y. No improvement
16^25^	M	Multiple (Neck, chest, and back)	None	Thinner dermis and a heavier density of elastic fibers and perivascular lymphohistiocytic infiltrate in the lesional skin compared to normal skin	None
1.75^20^	M	Single (Lower back)	None	None	None
39^26^	M	Multiple (Shoulders and back)	None	Increased melanin in the basal cell layer and thickened, tightly packed collagen bundles in the dermis	None
45^27^	M	Multiple (Trunk, arm, abdomen, back, and chest)	None	Slightly atrophic epidermis and markedly thickened collagen bundles. Slight mononuclear cell infiltrations around vessels and dermal appendages. Thinner dermis compared with normal adjacent skin	None
38^16^	F	Multiple (Leg, arm, and face)	Positive antinuclear antibody titer 1:320	Attenuated thickness of the dermis compared with the unaffected skin, decreased dermal papillae, and flattened rete pegs. Slight angioplasia and fibroplasia without inflammation	Hydroxychloroquine 400 mg daily. Minimal improvement after 5 mo. Facial and truncal lesions resolved after 1 yr Lesions on extremities remained as shallow depressions
18^12^	M	Multiple (back)	Antinuclear antibody titer 1:320 Imaging: sonographical dermal thickness in the involved skin was decreased by 46‐55% and hypodermis decreased by 10‐18% at the measured sites. The echogenicity of the skin in involved areas was almost the same as that of the normal skin	None	None
17^10^	F	Multiple (Abdomen, buttock, and back)	None	Indurated dermis with hyalinized and swollen collagen bundles. Collagen bundles were tightly packed in some areas. The elastic fibers, mostly in the upper dermis, were fragmentized, and the changes were better observed by orcein staining	None
22^17^	M	Multiple (Trunk, chest, abdomen, and back)	Negative rheumatoid factor and negative antinuclear antibody	Normal epidermis, mild periadnexal lymphohistiocytic inflammation, the absence of lichenoid tissue reaction, and mild thickening of collagen bundles. Direct immune‐fluorescence staining showed nonspecific weak segmental granular deposits of C3 at the dermoepidermal junction	Successive trials of midpotency topical corticosteroids, topical antifungals with no improvement. Improvement noted with successive trials of Q‐switched alexandrite laser, 755 nm, at 6‐wk intervals
65^28^	F	Single (Back)	Routine laboratory examinations were negative.^8^	Decreased dermal thickness and interstitial edema	None
14^29^	M	Multiple (Back and abdomen)	Elevated ESR and positive ANA 1:160. Liver and kidney function, urinalysis, electrolytes, and peripheral blood cells were normal. Anti‐Scl‐70 antibody and rheumatoid factor were negative	Thickening of collagen bundles in the mid to deep dermis in the involved lesion but not in the uninvolved skin. No inflammatory cell infiltrates	Topical steroids ineffective
18^30^	M	Multiple (Abdomen and back)	Negative titer for Lyme disease. Imaging: MRI showed no subcutaneous atrophy	Normal‐appearing epidermis with increased pigmentation in the basal layer	None
13^31^	F	Multiple (Back)	Antibody titer to double‐stranded DNA of 88 units (normal: 70 units). Serologic Studies for Borrelia burgdorferi antibodies is positive	Microscopic examination of a specimen from the involved skin showed a normal‐appearing epidermis but increased pigmentation in the basal layer	None

There are limited reports available in the literature with regards to treatment, and none have been found to be consistently effective for APP. There have been reports of tetracyclines utilized to prevent appearance of new lesions in patients who tested positive for the IgG antibody to B. burgdorferi.^13^, ^14^ In a retrospective study of 25 patients treated with penicillin (2 million IU/d) or 500 mg oral tetracycline three times daily, for 2‐3 weeks, showed clinical improvement in 20 patients.^15^ Another approach utilized hydroxychloroquine and topical corticosteroids which provided a good response in patients who also had lupus. Topical calcineurin inhibitors were found to have variable responses.^16^ Q‐switched alexandrite laser has also resulted in some clinical improvement of hyperpigmented lesions.^17^

The differential diagnosis includes Linear Atrophoderma of Moulin (LAM) and morphea, where distinctions need to be made to provide a diagnosis to the patient (Table 2). LAM has typically been reported to have an earlier onset and a distribution following Blaschko's lines. Similar to IAPP it also lacks inflammation. Although LAM, usually has an earlier onset, cases of congenital IAPP have been identified and blaschkoid IAPP, making the final diagnosis challenging.^18^, ^19^

**Table 2:** Differentiating Idiopathic atrophoderma of Pasini and Perini (IAPP) from Linear atrophoderma of moulin (LAM) and morphea

Characteristic	IAPP	LAM	Morphea
Age of onset	Onset between 20‐40 y of age. Longer disease course (1‐2 decades)	Onset between 6‐20 y of age	Disease course of active lesion (5 y)
Distribution	Commonly affects trunk and then progresses to include the chest, arms, and abdomen	Unilateral distribution following Blashko's lines	Single or multiple well‐defined plaques most commonly. Can present in linear distribution
Histopathology	Lacks inflammation. Decreased or clumped elastic fibers. Thinned dermis. Total amount of disaccharide per skin punch biopsy and the amount of DeltaDi‐4S(DS) low	Lacks inflammation	The presence of inflammation. Induration of lesion and epidermal atrophy. Alteration of the appendageal structures. Total amount of disaccharide per skin punch biopsy and the amount of DeltaDi‐4S(DS) increased

Differentiation from morphea has been difficult to establish. Some believe it could be on the spectrum of disease or others indicate IAPP could be the result after burn out from a previously inflammatory lesion.^22^ However, with more evidence in the literature, it has been shown to be a distinct entity, characterized by the lack of induration and epidermal atrophy, as well as cutaneous glycosaminoglycans different from those seen in morphea.^22^

## 4 CONCLUSION

Idiopathic Atrophoderma of Pasini and Pierini is a rare skin disease thought to affect dermal collagen organization and resulting in dermal atrophy. There is little evidence regarding precipitating factors available and the distinction between LAM and morphea results in a diagnostic challenge. In this case report, we described another case of IAPP in a 23‐year‐old man presenting with an asymptomatic atrophic hyperpigmented plaque on the lower back with normal laboratory results.

## CONFLICT OF INTEREST

The author(s) declared no potential conflicts of interest with respect to the research, authorship, and/or publication of this article.

## AUTHOR CONTRIBUTION

AM: wrote the introduction and the discussion. AJ: wrote the case presentation. MR: wrote the pathology section. OR: reviewed the manuscript and added the abstract.
